# Physiological determinants of heart rate responses and bout-to-bout stability during 2v2 and 4v4 small-sided games in football players

**DOI:** 10.3389/fpubh.2026.1766471

**Published:** 2026-01-16

**Authors:** Aleksandra Kisilewicz, Małgorzata Smoter, Robert Trybulski

**Affiliations:** 1 Faculty of Medicine, Wrocław University of Science and Technology, Wrocław, Poland; 2 Department of Basic Physiotherapy, Gdańsk University of Physical Education and Sport, Gdańsk, Poland; 3 Medical Department, Wojciech Korfanty Upper Silesian Academy in Katowice, Katowice, Poland; 4 Provita Żory Medical Center, Żory, Poland

**Keywords:** health monitoring, heart rate, performance, soccer, sports physiology, sports training

## Abstract

**Purpose:**

Injury prevention in sport is increasingly recognized as a health priority, and monitoring internal load during routine training offers a pragmatic pathway to reduce overuse risk and support long-term athlete health. This study examined the extent to which three practical physiological markers (resting heart rate (HRrest), submaximal warm-up heart rate after 60-s recovery (HRR), and Yo-Yo IR1 HRmax) explain mean heart rate (HRmean) and bout-to-bout variability (CV%) during two commonly used small-sided game (SSG) formats (2v2 and 4v4) in football players.

**Methods:**

Forty male amateur players completed HRrest, HRR, and Yo-Yo IR1 testing, followed by randomized, counterbalanced 2v2 and 4v4 SSG sessions (3 × 3-min bouts). HRmean and CV% were computed for each format. Associations were assessed using Pearson correlations and multiple linear regression.

**Results:**

HRmean did not differ significantly between 2v2 and 4v4 (166.9 ± 5.1 vs. 165.3 ± 6.0 bpm; t₃₉ = 1.35, *p* = 0.19), whereas CV% was lower in 2v2 (2.05 ± 1.16%) than 4v4 (2.85 ± 1.43%; t₃₉ = −2.52, *p* = 0.016). In 2v2, no physiological marker correlated with HRmean (all *p* > 0.30). In 4v4, HRR showed a moderate significant correlation with HRmean (*r* = 0.36, *p* = 0.023). Regression models explained 3.1% (2v2) and 16.3% (4v4) of HRmean variance, with HRR emerging as the only significant predictor in 4v4 (*β* = 0.20, *p* = 0.047). In a repeated-measures linear mixed-effects model (random intercept for player; fixed effect for format), the resting HR × format interaction for CV% was significant (*p* = 0.016).

**Conclusion:**

HRR is the sole physiological marker meaningfully associated with internal load during SSGs (and only in 4v4) while neither HRmean nor CV% in 2v2 is explained by the tested markers. More constrained formats (2v2) produce more stable cardiovascular responses. By identifying simple heart-rate–based indicators and game formats that yield more stable cardiovascular responses, these findings can inform scalable load-management strategies that help mitigate maladaptive overreaching and contribute to broader injury-prevention efforts in athletic populations, although future studies are necessary to confirm such hypothesis.

## 1 Introduction

Effective monitoring of internal training load is central to optimizing performance and minimizing bad overreaching in team sports, with heart rate (HR)–based measures among the most frequently used tools in professional football scenarios (1). HR indices collected at rest, during exercise, and in recovery provide integrative information about fitness, fatigue, and endurance performance, and are widely regarded as core components of contemporary athlete monitoring systems (2). Also resting HR can be classified as a marker that may reflect both cardiorespiratory fitness status and short-term readiness/fatigue state, acknowledging that its interpretation is context-dependent and influenced by recovery, illness, and non-training stressors (2). Small-sided games (SSGs) are constrained, drill-based forms of play (3) that allow coaches to simultaneously target technical–tactical behaviors and high cardiovascular loading in a time-efficient manner (4). Experimental studies has shown that appropriately designed SSGs can elicit HR responses comparable to or even exceeding those induced by intermittent running protocols or official match play, reinforcing their use as a conditioning stimulus (5). At the same time, repeated-measures studies reveals substantial within- and between-player variability in HR responses during standardized SSG protocols, indicating that players may experience different internal loads despite sharing identical task constraints (6). Recent syntheses indicate that internal-load measures (e.g., HRmean, %HRmax) generally show low variability compared with external-load and technical outcomes, and that it is important to distinguish within-session bout-to-bout stability from between-session reliability when interpreting variability magnitudes (4). Although HR-derived indices are not injury outcomes, internal-load monitoring is a core component of contemporary workload–response frameworks in which inappropriate load progression and inadequate recovery are theorized to contribute to maladaptation and injury risk (7, 8). In applied football context, HR-based internal-load information from SSGs is commonly used to (i) verify whether the intended cardiovascular stimulus was achieved, (ii) individualize exposure via bout number/duration and player rotations, and (iii) identify disproportionate responses (higher-than-expected HR for a given constraint set) that may indicate transient fatigue, heat strain, illness, or reduced readiness and therefore warrant altered training content or recovery emphasis (2).

Manipulating SSG format, particularly pitch size and number of players, significantly alters HRmean, rating of perceived exertion, and locomotor demands, with smaller formats generally associated with higher internal and external loads (9). For example, comparisons across formats consistently show that 2v2 configurations elicit higher HRmean and perceived exertion than larger formats such as 4v4, while also modifying technical actions and ball involvement patterns (10). Recent data in youth soccer further indicate that HR responses during 2v2 and 4v4 SSGs are closely related to technical performance indicators such as lost balls and successful shots, underscoring the sensitivity of HRmean to contextual task constraints (11). Beyond average intensity, within- and between-player variability in HR across repeated SSG bouts, often quantified using the coefficient of variation (CV%), has emerged as an importante marker for assessing the stability and responsiveness of different SSG formats (12). CV% provides an absolute index of within-player variability (typical error expressed relative to the mean) and is commonly used to quantify reliability/consistency of repeated physiological measures (13). Longitudinal work in professional football shows that internal and external load measures exhibit considerable session-to-session fluctuations during preseason and in-season periods, reinforcing the need to understand factors that influence the consistency of HR responses in SSGs (14).

At the individual level, resting HR is inversely associated with cardiorespiratory fitness and positively associated with cardiovascular risk, and typically declines in response to chronic endurance training (15). Cross-sectional comparisons between endurance-trained and untrained adults demonstrate that athletes display lower resting HR together with higher maximal oxygen uptake, supporting the use of resting HR as a global index of aerobic conditioning (16). In team sports, conceptual frameworks highlight that resting and exercise-related HR measures, along with heart rate variability, provide non-invasive insights into autonomic balance and aerobic fitness, and can be embedded within multivariate monitoring systems (17). Standardized submaximal fitness tests have gained popularity because exercise HR recorded at a fixed submaximal intensity shows high reliability and large inverse correlations with endurance performance tests in team sport athletes (18). Longitudinal observations indicate that changes in submaximal HR during running or shuttle-based protocols are sensitive to training-induced alterations in aerobic capacity in professional players, supporting their use for routine status monitoring (19). Nevertheless, recent surveys of load-monitoring practice in elite football emphasize substantial heterogeneity in the implementation and interpretation of HR-based indices, and point to a lack of evidence on how simple measures such as resting HR and submaximal HR relate to HR responses in football-specific SSG formats (20).

The Yo-Yo Intermittent Recovery (YYIR) tests are established field assessments of high-intensity intermittent running capacity in team sports and show strong associations with external running demands and high-intensity activity during match play (21). Foundational work on the YYIR tests demonstrated large correlations between YYIR performance and high-speed running distance in football, confirming their ecological validity for intermittent sport demands (22). Subsequent studies in youth soccer players have confirmed that YYIR1 exhibits good test–retest reliability and can discriminate between performance levels and positional roles (23). YYIR performance is also associated with HR-derived indices of match intensity, including time spent in high-intensity HR zones and post-exercise HR recovery, indicating that YYIR outcomes reflect integrated cardiovascular and neuromuscular function during intermittent play (24). Emerging evidence links YYIR results with technical and tactical performance in small-format games such as 2v2, suggesting that players with higher intermittent running capacity may better tolerate the demands of constrained SSG scenarios (25). Nonetheless, recent analyses emphasize that YYIR performance is influenced by multiple morphological and neuromuscular factors and do not clarify how YYIR levels compare with resting HR and submaximal HR in explaining HRmean and its stability across repeated SSG bouts of different formats (26).

Current evidence establishes SSGs as powerful yet variable training tools and confirms that HR-based markers (including resting HR, submaximal HR, and YYIR performance) each capture partially distinct aspects of aerobic fitness and intermittent exercise tolerance, while leaving their relative contributions to explaining HR responses during SSGs largely unresolved (27). The stability of HRmean across repeated SSG bouts, reflected by lower CV% in HR responses, is increasingly recognized as a key property for using SSGs to monitor training-induced aerobic adaptations, yet the physiological determinants of maintaining low HR variability over repeated 2v2 and 4v4 formats remain nuclear (28). In this context, stability refers specifically to within-session bout-to-bout consistency of HRmean (i.e., lower CV% across the three bouts), which is typically interpreted as a more predictable cardiovascular stimulus under standardized constraints, however low CV% should be interpreted pragmatically (predictable internal-load delivery) rather than as a proxy for superior adaptation per se. Meta-analytic work on sided-games further shows that exposure and intra-individual reliability of external high-speed and sprint running loads are strongly influenced by game format and constraints, reinforcing the need for format-specific analyses of internal load stability (29). However, HR responses during SSGs are multi-determined by task constraints (rules, area per player), game dynamics (ball-in-play time, transitions), and psychophysiological factors (competitive arousal and pacing) (4), so physiological markers may explain only a modest fraction of variance in HR outcomes.

Against this background, and in line with calls to integrate simple HR-derived markers into practical monitoring frameworks, it is pertinent to determine which HR-based measures best explain the internal load experienced during standardized SSGs (30). Therefore, the main aim of the present study was to identify which of three readily obtainable measures (resting HR, HR recovery post 60-s of a standardized warm-up running test, and HRmax at YYIR test level) best explains HRmean during 2v2 and 4v4 SSGs in football players, and a secondary aim was to determine which of these measures is most closely associated with the capacity to maintain lower CV% in HR responses across repeated SSG bouts and whether these relationships are format dependent, thereby providing format-specific guidance for integrating simple HR markers into SSG-based monitoring and prescription.

## 2 Methods

### 2.1 Experimental approach

**Figure 1:**
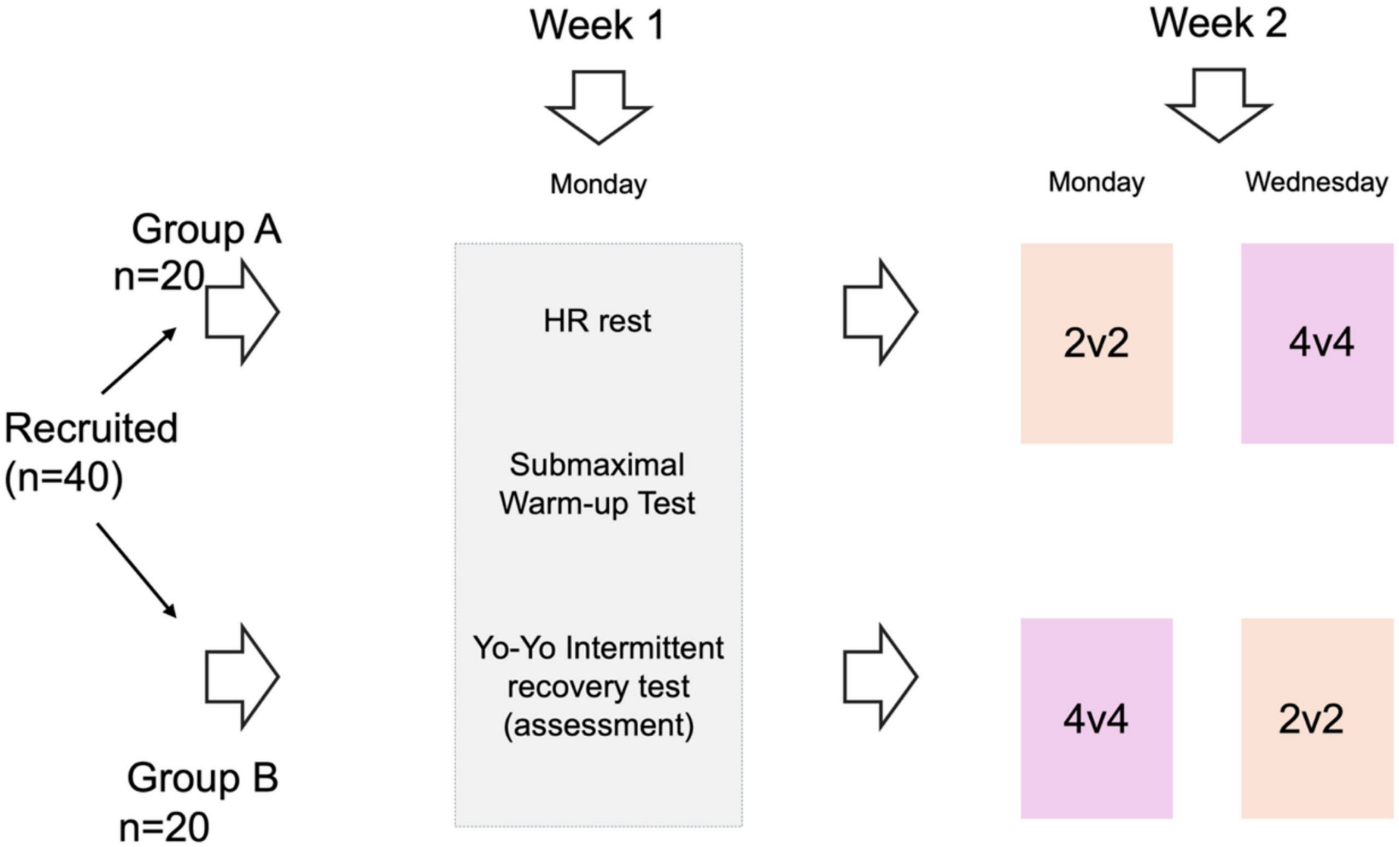
Illustration of the study design. HR, heart rate.

This study employed a randomized, counterbalanced crossover design conducted during the first two weeks of the in-season period. All procedures were carried out on outdoor natural-grass training pitches used routinely by the participating teams. Players were recruited from two amateur senior male football teams competing in regional leagues. Training loads and competitive schedules were maintained as usual, except for the testing sessions described below, which were integrated into each team’s regular training microcycle to ensure ecological validity. During Week 1, participants completed three baseline assessments in the same order: (i) resting heart rate (HRrest), (ii) heart rate post 60-s of a standardized submaximal warm-up running protocol (HRR), and (iii) the Yo-Yo Intermittent Recovery Test Level 1 (Yo-Yo IR1). HRrest was recorded in the morning under controlled conditions (supine position, minimal external stimuli), while the submaximal warm-up protocol and Yo-Yo IR1 were performed during regular training hours. During Week 2, all players completed two SSG formats (2v2 and 4v4) in single sessions separated by 48 h. Both sessions were scheduled within each team’s regular microcycle (Monday and Wednesday), and format order was counterbalanced across players. Therefore, any systematic microcycle-day effects were balanced between formats. The outcomes should be interpreted in light of match-day proximity and preceding training load, which were not quantified directly in this study. To control for order effects and reduce potential carryover bias, participants were randomly allocated (computer-generated block randomization stratified by team) to one of two sequences: (i) 2v2 on Monday followed by 4v4 on Wednesday; or (ii) 4v4 on Monday followed by 2v2 on Wednesday. This approach (Figure 1) constitutes a randomized, counterbalanced crossover design, ensuring that all players experienced both formats while balancing sequence effects across the sample.

Each SSG session consisted of three repeated bouts performed under standardized instructions, pitch dimensions, work-to-rest ratios, and timing. All sessions for both formats were scheduled at the same time of day for each team to reduce circadian influences on heart rate and performance. Players were instructed to maintain normal dietary and hydration habits, refrain from strenuous exercise 24 h prior to each test, and avoid caffeine or stimulant intake on testing days.

### 2.2 Participants

A total of 40 male amateur-level football players were enrolled in the study. All participants were active members of local amateur teams engaged in regular structured training (four sessions a week) and match play within regional league competitions. The group consisted exclusively of healthy adult players who were free from any known cardiovascular, metabolic, neurological, or musculoskeletal disorders that could interfere with maximal exercise performance. The players had a mean age of 25.0 ± 4.6 years, a mean height of 178.0 ± 6.4 cm, and a mean body mass of 74.5 ± 6.2 kg. Eligibility for participation required that athletes be male, between 18 and 40 years of age, and currently active in amateur football with at least two years of playing experience and consistent weekly training.

Participants were required to be free from injury for a minimum of six weeks prior to the assessments and capable of completing high-intensity field-based tests. Players were not included if they reported any condition that could contraindicate maximal exercise, if they used medications known to influence heart rate responses, if they presented with acute illness at the time of testing, or if they did not comply with the standardized pre-test preparation guidelines. Recruitment was conducted directly through collaboration with team coaches, and all players who fulfilled the eligibility criteria and agreed to take part were enrolled sequentially.

*A priori* sample size estimation was performed using GPower (Universität Düsseldorf, Germany), assuming a paired-samples design comparing cardiovascular responses between 2v2 and 4v4 formats as the primary analysis and a secondary focus on correlations between physiological markers and mean heart rate. Based on previous small-sided game literature reporting small-to-moderate differences in heart rate variability between formats and moderate associations (r ≈ 0.40–0.50) between physiological fitness markers and internal load, we set a minimum relevant effect size of Cohen’s d = 0.45 for paired comparisons of HR CV% and r = 0.45 for correlations between physiological markers and HRmean. With a two-sided *α* = 0.05 and statistical power of 80%, the required sample size was estimated at 34 players for the paired-samples t-test and 38 players for detecting the target correlation.

Prior to taking part in the study, all athletes—and when relevant, their legal guardians—were provided with comprehensive information regarding the purpose of the research, the testing procedures involved, and any potential risks or expected benefits. Each participant subsequently signed an informed consent form. The research was conducted in accordance with the ethical standards outlined in the Declaration of Helsinki and received approval from the Ethical Committee of the University of Physical Education and Sport in Gdańsk (approval number 14/3/2025). Participants were reminded that their involvement was entirely voluntary and that they could discontinue participation at any time without affecting their sporting activities. Throughout the duration of the study, no injuries or adverse events were recorded.

### 2.3 Small-sided games

The SSG sessions were performed during regular team training on outdoor natural-grass pitches and were preceded by the standardized FIFA 11 + warm-up program. Because FIFA 11 + can elicit a meaningful physiological load and elevate HR prior to subsequent tasks, a 3-min passive recovery was provided before the first bout. Importantly, the warm-up was identical across formats and the format order was counterbalanced, limiting systematic bias in between-format comparisons.

In our protocol, the 2v2 format was played on a 20 × 15 m field (area = 300 m^2^; 75.0 m^2^·player^−1^; width:length ratio = 0.75), while the 4v4 format was played on a 28 × 21 m field (area = 588 m^2^; 73.5 m^2^·player^−1^; width:length ratio = 0.75). These dimensions ensured an equivalent player/area ratio between formats (difference < 3%) and preserved the same width-to-length proportion, while remaining within the dimensional ranges and relative areas typically reported for small-to-moderate sided formats in high-level football (31, 32). This design choice was intended to isolate the effect of player number while controlling for two major determinants of internal load (relative area per player and pitch geometry) thereby improving interpretability of format comparisons in HR outcomes.

Each SSG session consisted of 3 bouts of 3-min interspersed with 2 min of passive recovery, and the total number of bouts was kept identical between 2v2 and 4v4 to equate total playing time. The use of short intermittent SSG bouts with brief recovery periods is consistent with previous work showing that formats such as 2v2–4v4 played in 2–4 min repetitions elicit high cardiovascular strain comparable to or exceeding official match demands, while allowing sufficient recovery to maintain intensity across repetitions (33).

Rules and task constraints were standardized across all sessions to isolate the effects of format and individual fitness measures on heart rate responses. Team composition was organized by the coaching staff to maintain positional balance and comparable playing level across opposing teams. Within each session, team assignments were kept constant across the three bouts. Both formats were played without goalkeepers using two small goals positioned centrally on each end line. Games followed the official laws of the game except for the offside rule, which was removed to encourage attacking play and maintain fluidity. The ball was immediately replaced by spare balls positioned around the pitch to minimize stoppages, and coaches provided consistent verbal encouragement to sustain effort. Verbal encouragement was delivered in a standardized manner (same coach per team, standardized frequency and wording cues delivered at regular intervals) to minimize psychophysiological differences attributable to encouragement. No limitations were imposed on the number of ball touches per player, and teams were instructed to play freely while attempting to score as many goals as possible. Across both formats, the same group of players, pitch surfaces, environmental conditions, and training schedule constraints were maintained, and all SSGs were embedded at the same point of the session (after the FIFA 11 + and a short rest) to minimize confounding effects of prior fatigue or circadian variation.

### 2.4 Resting heart rate assessment

Resting heart rate (HRrest) was assessed using a standardized supine protocol (34), in which resting HR was defined as the lowest value recorded while players lie in a supine position for 10 min. All measurements were performed in the first week of the in-season period, in a quiet room adjacent to the training facility, under stable environmental conditions. Players were tested individually on non-training mornings, between 10:00 and 11:00, and were instructed to avoid strenuous exercise, alcohol, and caffeine for 24 h before the test and to consume only a light meal at least 2 h prior to arrival, in line with standard recommendations for resting heart rate and heart rate variability assessments (35).

Upon arrival, each player was fitted with a Polar H10 chest-strap heart rate sensor (Polar Electro Oy, Kempele, Finland), positioned horizontally at the level of the xiphoid process according to the manufacturer’s instructions, ensuring full electrode–skin contact after moistening the contact area with water. Beat-to-beat R–R interval data were recorded continuously for 10 min while the player lay supine on a treatment table, breathing spontaneously and refraining from talking or any voluntary movement. HRrest was operationally defined, as the lowest heart rate value observed during the 10-min supine recording, and this value was retained for subsequent analyses as the individual’s resting heart rate.

The Polar H10 device was selected because ECG-validated studies have shown that its R–R interval and heart rate data at rest demonstrate near-perfect agreement with a 12-lead electrocardiogram, supporting its use as a criterion-level tool for resting heart rate and heart rate variability measurement in sport and clinical contexts (36). Raw R–R series were exported for each player and inspected using the recording software’s automatic artefact detection. Segments containing obvious signal loss or non-physiological spikes were excluded, but no additional smoothing or averaging beyond the 10-min window was applied, to remain faithful to the original protocol definition of HRrest as the lowest supine value over that period.

### 2.5 Sub-maximal warm-up protocol

The submaximal warm-up running test (SWT) used in the present study followed exactly the validated and reliable protocol (37), which has demonstrated very high test–retest reliability for exercise heart rate and strong validity for monitoring high-intensity intermittent running fitness in professional soccer players. All SWT assessments were conducted during Week 1, before the technical–tactical portion of each training session, and on day following 48 h of rest to avoid residual fatigue. The SWT was performed on a regulation soccer pitch with two parallel lines placed 100 m apart, exactly matching the configuration described in the foundational validation study (37). Players were instructed to run continuously back and forth between the two lines for 4 min at a fixed speed of 12 km·h^−1^, with a change of direction every 100 m. Running speed was controlled by an auditory pacing signal, identical to the original protocol (37), to ensure uniform intensity across participants and sessions.

Heart rate was recorded continuously at 1-s intervals throughout the entire test using the Polar H10 chest-strap sensor (Polar Electro Oy, Kempele, Finland). HR was calculated as the mean HR during the final 30 s of the 4-min running phase (i.e., between 3:30 and 4:00), following the validated procedure (37). After completing the 4-min continuous run, players were required to stop immediately and stand still for 1 min to allow measurement of heart rate recovery (HRR). Heart rate recovery was quantified during the first 60 s of passive standing recovery (4:00–5:00) using the HRpost1 (the mean HR during the 1-min recovery, i.e., HRR during recovery). The measures have been shown to exhibit high reliability (ICC = 0.83–0.95) and excellent sensitivity to fitness status changes in professional soccer players (37). This SWT protocol was selected because it is short in duration (5 min total), requires minimal setup, fits naturally into the early portion of a football warm-up, and has been shown to produce highly reproducible HR values (CV = 1.4%), making it a robust tool for monitoring aerobic and intermittent running fitness in applied football environments.

### 2.6 The Yo-Yo intermittent recovery test

Intermittent aerobic performance was evaluated using the Yo-Yo Intermittent Recovery Test Level 1 (Yo-Yo IR1), a well-established field assessment designed to quantify an athlete’s ability to tolerate and repeat high-intensity running efforts interspersed with short recovery periods. The test has been extensively validated in intermittent team sports (22), particularly soccer, where it has shown strong relationships with competition level, seasonal fitness changes, and match running demands. Players with a higher competitive profile typically achieve superior Yo-Yo IR1 distances, and improvements in test outcomes have been shown to mirror gains in maximal oxygen uptake and match-related high-intensity actions (38). Moreover, test–retest research in young and adult soccer populations consistently demonstrates excellent reproducibility of Yo-Yo IR1 performance, with intraclass correlation coefficients generally falling between 0.87 and 0.98 and coefficients of variation typically below 8%, supporting its appropriateness for longitudinal monitoring (39).

All Yo-Yo IR1 assessments were conducted one week before the start of the SSG intervention phase. Testing occurred on outdoor synthetic surfaces at each club’s regular training venue, within the same early-evening period used for the SSG sessions to minimize circadian influences. Before the test, players completed the sub-maximal warm-up test. All attempts were supervised by the research team using standardized instructions and verbal encouragement to ensure maximal effort.

The Yo-Yo IR1 protocol consists of repeated 2 × 20 m shuttle runs paced by an audio signal, with running speed progressing incrementally as the test advances. After each shuttle, players complete a 10-s active recovery period involving 2 × 5 m of low-intensity jogging, following the original methodological guidelines (38). Participants begin behind the starting line and must reach each turning point precisely in time with the sound cues. The test is terminated once a player twice fails to reach the designated line at the correct time or stops voluntarily. The maximum heart rate reached during the test was recorded for subsequent statistical analysis.

In the present study, we used the maximal heart-rate response attained during the Yo-Yo test as an HR-based ‘ceiling’ marker rather than as a direct index of intermittent running capacity (39). HRpeak obtained during Yo-Yo protocols has shown good test–retest reliability, with small typical measurement error for heart-rate responses in youth football cohorts, supporting its use as a practical maximal reference value in field settings (23). From an applied monitoring perspective, an individually measured HRmax/HRpeak is also preferred over age-predicted equations in soccer players, and it provides a physiologically meaningful anchor for interpreting HR-derived internal-load outcomes (40).

### 2.7 Heart rate monitoring

Heart rate responses during all SSGs sessions were captured using the Polar Team Pro system (Polar Electro Oy, Kempele, Finland), which integrates chest-strap ECG-based sensors with a team-level data acquisition platform. Each player was equipped with their individual HR strap approximately 10 min before the standardized warm-up to ensure stable electrode contact and enable the system to detect any connectivity issues before the session began. Heart rate was recorded at a frequency of 1 Hz during the entire session, including the warm-up, all 3-min SSG bouts, and the intervening passive rest periods. Players were instructed to position the strap consistently across sessions, and research staff verified proper device operation prior to each data collection period.

For each bout, mean heart rate (HRmean) was calculated as the average of all valid heart rate samples obtained during the 3 × 3 min active playing phase, with recovery periods excluded from the computation. HRmean from each bout represented the internal load for that specific repetition. For each SSG format, the session-level internal load was derived by averaging HRmean values across the three bouts performed. To evaluate the stability of cardiovascular load across repeated bouts, the coefficient of variation (CV%) of HRmean was determined for each player and format. CV% was computed as: CV% = (SD of HRmean across the 3 bouts / Mean HRmean across the 3 bouts) × 100.

### 2.8 Statistical analysis

Descriptive statistics are reported as mean ± standard deviation (SD) with minimum and maximum values; for skewed variables, median (interquartile range) is additionally provided. Distributional characteristics were assessed using Shapiro–Wilk tests and Q–Q plots. Model assumptions were evaluated using residual-versus-fitted plots (homoscedasticity) and residual diagnostics. Potential outliers were inspected using standardized z-scores and boxplots; no data imputation was performed. Multicollinearity among predictors was examined using variance inflation factors (VIF).

To compare cardiovascular responses between formats, paired-samples t-tests were applied to session-level HRmean (2v2 vs. 4v4) and HR CV% (2v2 vs. 4v4). Effect sizes were quantified using Cohen’s d for paired samples with 95% confidence intervals (CI).

Associations between each physiological predictor (resting HR, warm-up submaximal exercise HR [HRR], and Yo-Yo HRmax) and the two outcomes (HRmean and CV%) were first examined using Pearson correlation coefficients. Ninety-five percent CIs for correlations were computed using Fisher’s r-to-z transformation. These correlations were performed separately for each SSG format (2v2 and 4v4) to support format-specific interpretation.

To evaluate the relative contribution of predictors within each format, multiple linear regression models were fitted separately for 2v2 and 4v4, with resting HR, HRex, and Yo-Yo HRmax entered simultaneously. Standardized coefficients, partial R^2^, and overall model F-tests were reported alongside predictor-level *p*-values. The same approach was applied with CV% as the dependent variable for each format. VIF values were inspected to confirm low collinearity across predictors.

To test whether predictor–outcome relationships differed by format, combined-format analyses used a repeated-measures framework. Each player contributed two observations (2v2 and 4v4) in a long-format dataset. For each outcome (HRmean and CV%), we fitted linear mixed-effects models with a random intercept for player to account for within-subject dependence. Fixed effects included format (dummy coded: 0 = 2v2, 1 = 4v4), the centered predictors (resting HR, HRR, Yo-Yo HRmax), and predictor × format interaction terms. Significant interaction terms were interpreted as evidence of format-dependent associations. Team (two teams) was included as a fixed effect. As sensitivity checks, robust (sandwich) standard errors were examined and log-transformation of CV% was explored; conclusions were unchanged and diagnostics supported the adequacy of model assumptions.

All analyses were performed in R (version 4.5.2; R Foundation for Statistical Computing, Vienna, Austria). Statistical significance was set *a priori* at *α* = 0.05, and 95% CIs are reported where appropriate.

## 3 Results

Across the sample, resting heart rate averaged 72.7 ± 8.3 bpm (range 58–84 bpm), HRR during the submaximal warm-up test was 145.1 ± 9.9 bpm (128–164 bpm), and the maximal heart rate recorded during the Yo-Yo IR1 test (HRmax) was 183.7 ± 5.4 bpm (174–194 bpm). Mean heart rate during 2v2 SSGs was 166.9 ± 5.1 bpm (155.0–176.3 bpm), whereas mean heart rate during 4v4 SSGs was 165.3 ± 6.0 bpm (152.0–176.6 bpm). The within-session coefficient of variation of HR across bouts was 2.05 ± 1.16% (0.35–5.07%) in 2v2 and 2.85 ± 1.43% (0.89–7.34%) in 4v4.

When comparing formats, mean HR was slightly higher in 2v2 than in 4v4, but the estimate was imprecise and did not reach statistical significance (166.9 ± 5.1 vs. 165.3 ± 6.0 bpm; mean paired difference *Δ* = 1.60 bpm, 95% CI − 0.80 to 4.00; t₃₉ = 1.35, *p* = 0.19, Cohen’s d = 0.21). In contrast, HR variability across bouts was significantly lower in 2v2 than in 4v4 (2.05 ± 1.16 vs. 2.85 ± 1.43%; mean paired difference Δ = −0.80, 95% CI − 1.44 to −0.16; t₃₉ = −2.52, *p* = 0.016, Cohen’s d = −0.40), indicating more stable cardiovascular responses across repeated bouts in the smaller format.

**Figure 2:**
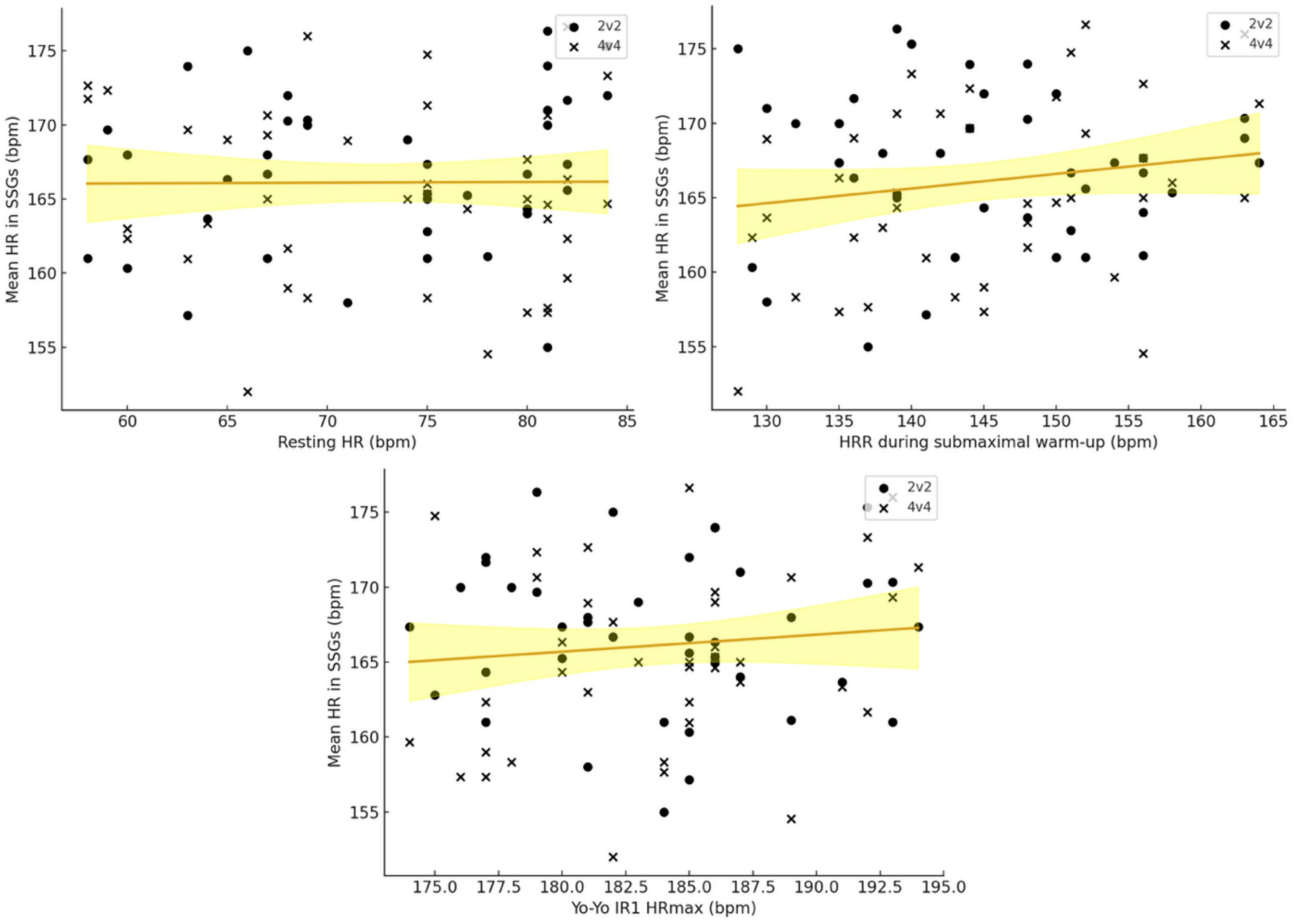
Regression plots illustrating the heart rate (HR) mean in both 2v2 and 4v4 format, and the resting HR, heart rate recovery in sub-maximal warm-up test (HRR), and the HRmax in Yo-Yo intermittent recovery test level 1.

Associations between the three physiological markers and mean HR during SSGs (Figure 2) were generally small to moderate. In 2v2, Pearson correlations between HRmean and resting HR (*r* = 0.17, *p* = 0.30), HRR (*r* = −0.04, *p* = 0.80) and Yo-Yo HRmax (*r* = −0.03, *p* = 0.86) were all trivial to small and non-significant. In 4v4, resting HR and Yo-Yo HRmax again showed non-significant relationships with HRmean (*r* = −0.13, *p* = 0.43 and *r* = 0.23, *p* = 0.16, respectively), whereas HRR displayed a significant positive association with mean HR (*r* = 0.36, *p* = 0.023; 95% CI 0.05–0.60). Thus, players who exhibited higher HR during the standardized submaximal warm-up tended to experience higher mean HR during 4v4, while no such relationship was evident in 2v2.

Multiple linear regression models confirmed these patterns. For 2v2, the combined model including resting HR, HRR and Yo-Yo HRmax explained only 3.1% of the variance in HRmean (R^2^ = 0.03, adjusted R^2^ = −0.05, overall *p* = 0.77), and none of the individual predictors reached significance (resting HR: *β* = 0.11 bpm·bpm^−1^, *p* = 0.31; HRR: *β* = −0.03 bpm·bpm^−1^, *p* = 0.77; Yo-Yo HRmax: β ≈ 0.00 bpm·bpm^−1^, *p* = 0.99). For 4v4, the same set of predictors accounted for 16.3% of the variance in HRmean (R^2^ = 0.16, adjusted R^2^ = 0.09, overall *p* = 0.090). In this model, HRR emerged as the only significant independent predictor of mean HR (*β* = 0.20 bpm·bpm^−1^, *p* = 0.047), whereas resting HR (*β* = −0.10 bpm·bpm^−1^, *p* = 0.38) and Yo-Yo HRmax (*β* = 0.13 bpm·bpm^−1^, *p* = 0.46) were not significant. Variance inflation factors for the three predictors were close to 1.0 in all models, indicating negligible multicollinearity. In the combined-format analysis, we fitted a linear mixed-effects model (random intercept for player) including format (2v2 vs. 4v4), the three centered predictors (resting HR, submaximal warm-up HRR, Yo-Yo HRmax), and predictor × format interaction terms. The model provided limited overall explanatory value (marginal R^2^ = 0.13; likelihood-ratio test vs. intercept-only model: *p* = 0.12). The only format-dependent association supported by the interaction terms was for HRR (HRR × format: *β* = 0.228 bpm·bpm^−1^, *p* = 0.049), indicating that HRR related positively to HRmean in 4v4 (*β* = 0.201 bpm·bpm^−1^, 95% CI 0.027 to 0.376, *p* = 0.024) but not in 2v2 (*β* = −0.026 bpm·bpm^−1^, *p* = 0.77). Interactions involving resting HR and Yo-Yo HRmax with format were not statistically significant (both *p* ≥ 0.12).

**Figure 3:**
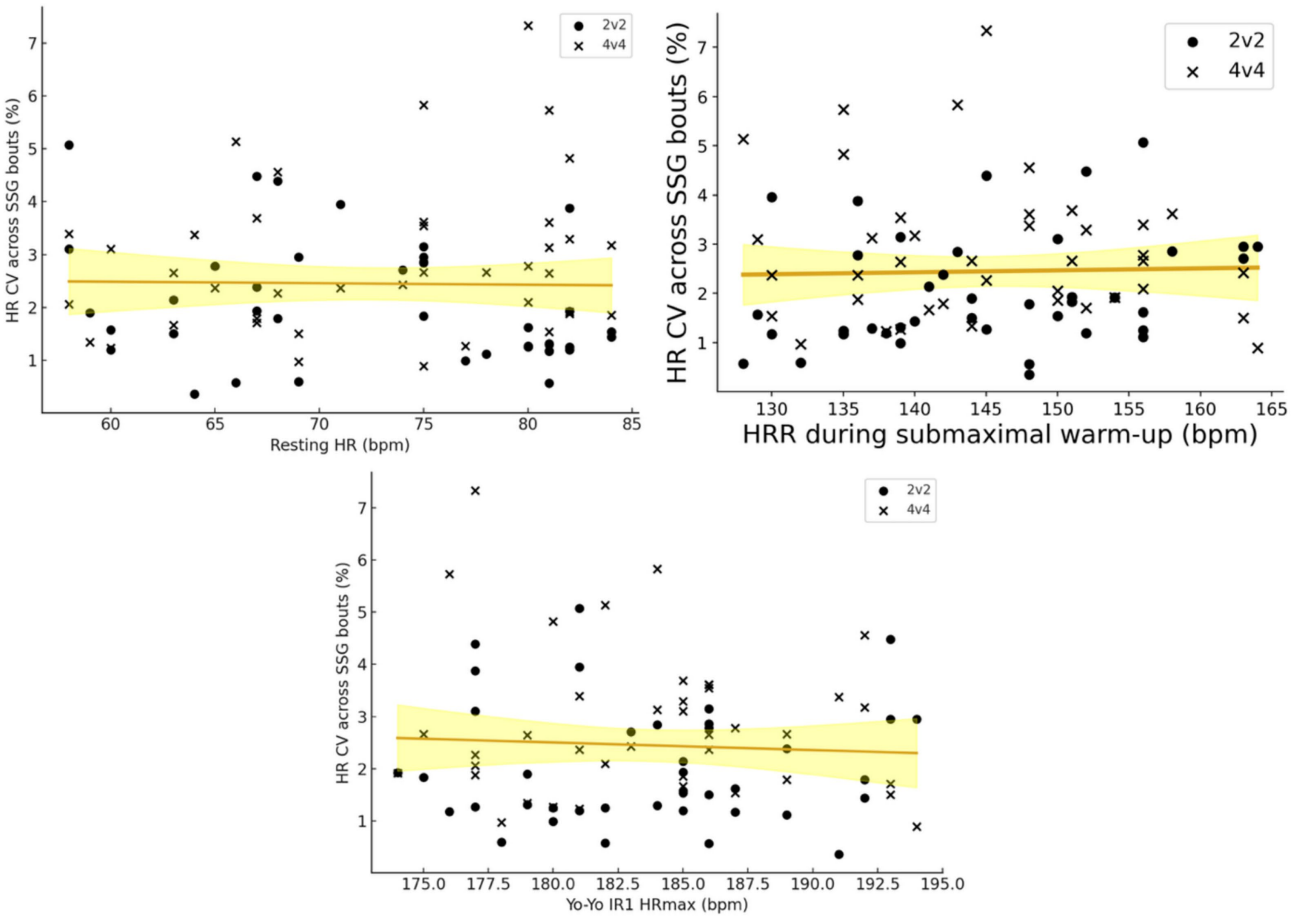
Regression plots illustrating the heart rate (HR) coefficient of variation over the bouts (%) in both 2v2 and 4v4 format, and the resting HR, heart rate recovery in sub-maximal warm-up test (HRR), and the HRmax in Yo-Yo intermittent recovery test level 1.

For the stability of cardiovascular load, correlations between the physiological markers and the CV% of HR across bouts were generally small, with only one borderline association (Figure 3). In 2v2, CV% showed a moderate, negative but non-significant correlation with resting HR (*r* = −0.30, *p* = 0.065), such that players with higher resting HR tended to exhibit slightly lower variability of HR across bouts. Correlations with HRR (*r* = 0.25, *p* = 0.12) and Yo-Yo HRmax (*r* = 0.02, *p* = 0.92) were small and not statistically significant. In 4v4, none of the predictors were significantly associated with CV% (resting HR: *r* = 0.21, *p* = 0.20; HRR: *r* = −0.15, *p* = 0.35; Yo-Yo HRmax: *r* = −0.12, *p* = 0.45).

Format-specific multiple regressions with CV% as the outcome were consistent with the correlation analyses. In 2v2, the model including resting HR, HRR and Yo-Yo HRmax explained 16.7% of the variance in CV% (R^2^ = 0.17, adjusted R^2^ = 0.10, overall *p* = 0.083), but none of the individual predictors reached conventional significance (resting HR: *β* = −0.06%·bpm^−1^, *p* = 0.16; HRR: *β* = 0.04%·bpm^−1^, *p* = 0.18; Yo-Yo HRmax: *β* = 0.02%·bpm^−1^, *p* = 0.43). In 4v4, the same predictors accounted for only 7.2% of the variance in CV% (R^2^ = 0.07, adjusted R^2^ = −0.00, overall *p* = 0.43), and again none of the individual coefficients were significant (all *p* ≥ 0.20).

To examine whether the relationships between physiological markers and HR variability differed by format, we fitted a linear mixed-effects model (random intercept for player) with CV% as the dependent variable, including format, the three centered predictors, and predictor × format interactions. The overall model supported modest explanatory value (marginal R^2^ = 0.19; likelihood-ratio test vs. intercept-only model: *p* = 0.020). The main effect of format was significant, with CV% higher in 4v4 than 2v2 by 0.80 percentage points at average predictor values (*β* = 0.799%, *p* = 0.0033), consistent with the paired t-test. The resting HR × format interaction was also significant (*β* = 0.081%·bpm^−1^, *p* = 0.016), indicating a more negative resting-HR slope in 2v2 (*β* = −0.045%·bpm^−1^, *p* = 0.059) and a small, non-significant positive slope in 4v4 (*β* = 0.036%·bpm^−1^, *p* = 0.13). In interpretive terms, a 10-bpm higher resting HR would correspond to 0.45 percentage points lower CV% in 2v2 (i.e., slightly greater within-session stability), whereas the analogous change in 4v4 would be ~0.36 percentage points higher CV% (a small, non-significant effect). Although these differences are modest in absolute terms, they are potentially meaningful for monitoring because they are of similar magnitude to typical within-session CV% values (2–3%). Interactions involving HRR and Yo-Yo HRmax did not reach conventional significance (*p* = 0.057 and *p* = 0.95, respectively).

**Figure 4:**
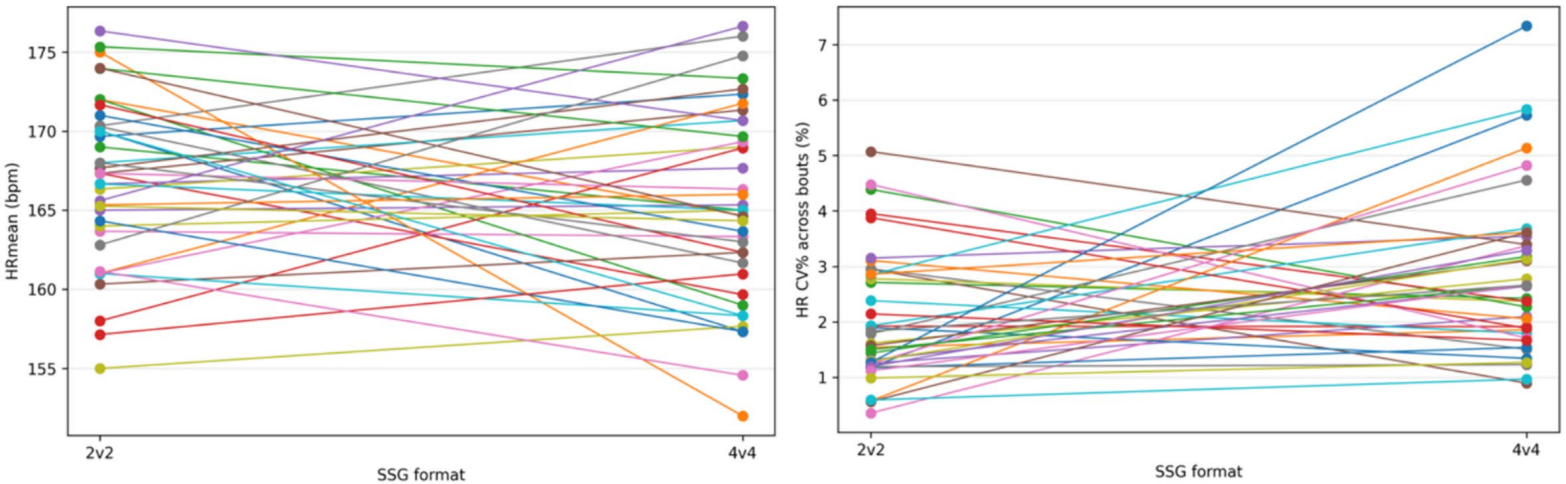
Within-player paired responses for HRmean and within-player paired responses for HR CV%.

To illustrate whether the negligible group-level difference reflects consistent individual responses or heterogeneous changes, Figure 4 present within-player paired-line plots for HRmean and HR CV% across the 2v2 and 4v4 formats.

## 4 Discussion

Recent research highlight that variability is intrinsic to SSGs, with internal-load measures (e.g., HRmean, %HRmax) typically showing lower variability than technical and high-speed external-load outputs, and with variability differing depending on whether it is quantified within-session (bout-to-bout) or between-session (day-to-day). Building on this literature, the present study focused specifically on within-session bout-to-bout stability (CV% across three repetitions) and tested whether commonly collected HR-derived markers (resting HR, HRR during sub-maximal warm-up test, and Yo-Yo HRmax) help explain individual differences in HRmean and stability during 2v2 versus 4v4 formats. The primary findings were that HRR was the only measure associated with HRmean, and this relationship emerged exclusively during 4v4. Resting HR and Yo-Yo IR1 HRmax showed no meaningful associations with HRmean in either format, and none of the markers showed strong explanatory capacity for HR variability across repeated bouts, although resting HR displayed a modest format-dependent pattern. A plausible explanation for the null findings for Yo-Yo HRmax is that peak HR in maximal field tests often clusters near an individual ceiling (limited between-player spread), whereas Yo-Yo performance outcomes (distance/level) or running speed at standardized physiological thresholds typically provide stronger discriminatory information about intermittent running capacity. Additionally, HR variability was systematically lower in 2v2 than in 4v4, indicating greater bout-to-bout cardiovascular stability in more constrained formats. Practically, lower CV% in 2v2 suggests that this format may be preferable when coaches aim to deliver a standardized cardiovascular stimulus or use HRmean as a routine monitoring readout (reduced within-session ‘noise’), whereas relatively higher CV% in 4v4 may be acceptable (or even desirable) when the goal includes exposing players to more variable tactical–contextual demands while maintaining a broadly similar mean internal load.

The finding that mean HR did not differ significantly between 2v2 and 4v4 parallels previous research showing that smaller-sided games can yield high cardiovascular responses, and that variation in format does not always translate to mean HR differences. For example, a study (5) found comparable cardiovascular loads across multiple SSG formats. In our data the lack of mean HR difference suggests that, from a cardiovascular-load viewpoint, both formats can be used interchangeably to elicit similar mean HR. A likely explanation is that although the number of players changed, other constraints (e.g., pitch size, bout/rest durations) were held constant, thereby preserving internal-load consistency. Coaches frequently manipulate player numbers, pitch size, and bout structure to target intensity (9). Therefore, our results reinforce that mean HR alone may not be sensitive to format changes when other variables are controlled. However, because area per player was closely matched across formats and SSGs were preceded by a standardized warm-up, HRmean may have been partially ‘ceilinged,’ reducing sensitivity to player-number changes.

In contrast, the significantly lower HR CV% in 2v2 suggests greater physiological response stability in that format compared to 4v4. This aligns with findings (6) who reported smaller within-player variability in HR in the 3v3 format compared to 5v5. The probable mechanism is that fewer players amplify involvement and intensity per individual and reduce heterogeneity of effort, thus leading to more homogeneous responses. In our 2v2 scenario each player is more consistently active, reducing drop-off periods, hence lowering CV. Thus, if coaches seek more reproducible cardiovascular stimulus across players and sessions, smaller formats (such as 2v2) may be preferable. A plausible explanation is that 2v2 increases individual involvement and reduces low-engagement phases, however, without ball-in-play time, technical involvement, or external-load tracking, this mechanism cannot be confirmed. For applied monitoring, interpretability will be highest when the SSG is performed at a consistent microcycle location (e.g., a comparable day relative to match day), time of day, and standardized warm-up. Using the observed within-session CV% (2–3%), practitioners may consider individual deviations in HRmean that exceed typical within-session ‘noise’ (e.g., several bpm beyond a player’s usual response under the same constraints) as potential flags for follow-up rather than as deterministic indicators of maladaptation.

The fact that only HRR (sub-maximal warm-up HR) predicted mean HR in the 4v4 format, explaining about 16% of the variance, while resting HR and Yo-Yo IR1 HRmax did not, merits discussion. Previous research has shown that aerobic fitness markers (such as HRmax or VO₂max) sometimes correlate with internal load during SSGs, but often the associations are weak or inconsistent (41). The limited predictive power might reflect that SSGs impose complex multifactorial demands (technical, tactical, motivational) rather than pure aerobic challenge (42). In our case, HRR may reflect immediate cardiovascular readiness or autonomic state prior to session, and thus correlate with how intensely the player engages physiologically during the bout. That this predictive association emerges only in the 4v4 (and not in 2v2) suggests that when format allows more variation in involvement (i.e., larger team size), pre-session physiological readiness becomes a more important determinant of cardiovascular load, whereas in highly constrained, high-engagement formats (2v2) the format’s intensity may override these individual differences. However, this association may also reflect coupling between submaximal exercise HR responsiveness and HR during game-based activity, rather than acute readiness per se.

It is also important to interpret the non-significant prediction of HR variability (CV%) by our physiological markers. Despite resting HR showing a format-interaction, no marker reliably predicted variability across formats. This suggests that variability of internal load in SSGs depends more on format constraints, game dynamics, player behavior and possibly motivation and tactics more than on baseline physiological status (43). Earlier work reported that while mean %HRmax responses were high and relatively stable (CV around 2.6–3.8%), other metrics such as RPE and blood lactate exhibited larger inter-player variability (44). Hence, practitioners should not rely solely on baseline physiological tests to anticipate variability. Rather, controlling session design (player number, area, bouts) is more important for managing variability.

Several limitations deserve acknowledgement. Generalisability is limited because the sample comprised two amateur teams from the same regional context. Training density, match congestion, and tactical/physical demands can differ substantially in elite environments, which may alter both HR responses to SSG constraints and the interpretability of HR-based monitoring signals. Our sample consisted of amateur male players, which may limit the applicability of our findings to elite or female populations. Although we manipulated number of players (2v2 vs. 4v4), other potential influences (such as pitch size per player, tactical instructions, ball-in-play time) were standardized and may nonetheless have influenced responses. Because CV estimates are sensitive to the number of repeated observations, CV% derived from three bouts should be interpreted as a pragmatic within-session stability indicator rather than a definitive reliability estimate. Moreover, because external-load measures (e.g., distance, accelerations, impacts) were not measured, similar HRmean across formats should be interpreted as similar cardiovascular strain only, not necessarily similar mechanical loading. Future research should manipulate these design variables. We used only heart rate metrics as internal load indicators, thus inclusion of external load measures (GPS, accelerometry) or perceptual measures (RPE) would offer a more complete picture.

From a coaching and sport science perspective, our findings suggest that measuring sub-maximal warm-up HR (HRR) may offer some predictive insight into cardiovascular load—particularly in 4v4 formats—but this predictive utility is limited. Given that HR variability (CV%) is lower in the 2v2 format, coaches seeking more reproducible cardiovascular stimuli across players and sessions might prefer 2v2 for certain training blocks. However, since mean HR did not differ between formats, both 2v2 and 4v4 may be used to elicit comparable average cardiovascular load. Ultimately, session design should emphasize format constraints (player number, pitch size, bout/rest durations) rather than relying solely on baseline physiological tests for regulating cardiovascular stimulus in SSG training.

## 5 Conclusion

This study examined whether three physiological markers (resting HR, submaximal warm-up HRR, and Yo-Yo IR1 HRmax) could explain individual differences in cardiovascular responses during two commonly prescribed small-sided game formats in football. Under the present protocol (2v2 vs. 4v4 with near-equal area per player), the specific heart-rate–derived markers examined (HRrest, HRR from a standardized submaximal test, and Yo-Yo IR1 HRmax) explained only a small-to-modest proportion of variance in SSG HR outcomes, and this explanatory value was format-dependent. Across both formats, mean HR was similar, yet 2v2 produced more stable bout-to-bout heart-rate responses than 4v4, indicating that game format exerts a stronger influence on HR variability than on average internal load. Among the physiological markers, only HRR demonstrated meaningful explanatory value, and exclusively in 4v4, where it accounted for a modest portion of HRmean variance. Resting HR and Yo-Yo IR1 HRmax did not predict mean HR or its variability in either format. These findings show that cardiovascular markers have limited utility for predicting internal load during small-sided games, and that their predictive capacity is format-dependent. The consistency of cardiovascular responses appears to be shaped more by structural constraints of the game (likely player number) than by baseline physiological status. Thus, practitioners should rely on careful manipulation of SSG design variables, rather than on pre-session physiological measures, when the objective is to control or anticipate cardiovascular load in football training. Although the present findings are performance-focused, improving the precision of day-to-day load management through SSG design may also have downstream relevance for athlete health by supporting injury-prevention and public-health–aligned objectives (e.g., minimizing excessive training stress and avoidable time-loss from sport).

## Data Availability

The raw data supporting the conclusions of this article will be made available by the authors, without undue reservation.
